# Risk of Non-Vertebral Fracture in Gout Compared to Rheumatoid Arthritis

**DOI:** 10.3390/jcm10204655

**Published:** 2021-10-11

**Authors:** Soo-Kyung Cho, Jun Liu, Yinzhu Jin, Seoyoung C. Kim

**Affiliations:** 1 Division of Pharmacoepidemiology and Pharmacoeconomics, Brigham and Women’s Hospital, Harvard Medical School, Boston, MA 02215, USA; skchomd@hanyang.ac.kr (S.-K.C.); jliu3@bwh.harvard.edu (J.L.); yjin9@bwh.harvard.edu (Y.J.); 2 Division of Rheumatology, Hanyang University Hospital for Rheumatic Diseases, Seoul 04763, Korea; 3 Division of Rheumatology, Immunology and Allergy, Department of Medicine, Brigham and Women’s Hospital, Harvard Medical School, Boston, MA 02215, USA

**Keywords:** gout, fracture, rheumatoid arthritis, cohort study

## Abstract

Objective: To evaluate the risk of non-vertebral fractures in patients with gout compared with those with rheumatoid arthritis (RA). Methods: Using claims data from Medicare (2008–2015), we conducted a cohort study of patients with gout versus RA matched on age, sex, and index date with a 1:1 ratio. The primary outcome was a composite endpoint of non-vertebral fractures including hip, pelvis, humerus, and wrist identified with the validated algorithms. We also assessed hip fractures separately. Multivariable Cox proportional hazards regression estimated the hazard ratio (HR) for the outcomes in gout versus RA adjusted for 45 covariates. Results: We included a total of 134,157 matched pairs of gout and RA patients (mean age: 73.7 years). Risk factors for fracture were more prevalent in RA, while other comorbidities including obesity, coronary heart disease, hypertension, and diabetes were more common in gout. Over the mean 2.8 years follow-up, the incidence rate (IR)/1000 person-year (PY) of non-vertebral fractures was 10.42 in gout and 15.01 in RA. For hip fractures, the IR/1000 PY was 4.86 in gout and 7.73 in RA. The multivariable HR associated with gout versus RA was 0.84 (95% confidence interval (CI) 0.80–0.88) for non-vertebral fractures and 0.76 (95% CI 0.71–0.82) for hip fractures. Stratified analyses by age, sex, prior fractures, steroid use, and TNF inhibitor use showed similar results. Conclusions: In this large cohort of older patients, gout was associated with a modestly decreased risk of non-vertebral or hip fractures versus RA. However, non-vertebral fractures occurred frequently in both gout and RA.

## 1. Introduction

Gout is a common inflammatory arthritis characterized by hyperuricemia leading to crystallization of monosodium urate in joints and other tissue [1,2]. The prevalence of gout increases with age and is 3 to 4 times higher in men than in women [3]. However, in women, the risk of gout increases sharply after menopause [4]. Gout is associated with an increased risk of comorbidities [5] and all-cause mortality as well as cardiovascular mortality [6]. The global burden of gout and associated comorbidities is substantial and has increased over the past decades [3].

It has been hypothesized that high serum urate levels would play a role in preserving bone mineral density (BMD) through its antioxidant effect [7]. Some studies report that high serum urate levels were associated with a reduced risk of incident osteoporotic fractures [8,9,10]. However, paradoxically, high serum urate levels may cause oxidative stress and its antioxidant effect becomes pro-oxidant [7], particularly when serum urate becomes supersaturated [11]. High serum urate levels could also cause an increase in the pro-inflammatory cytokines that promote bone resorption and inhibit bone formation [12,13,14,15]. Furthermore, during gout flare, an acute local inflammatory reaction of monocytes and macrophages with monosodium urate crystals occurs, leading to the release of interleukins (ILs) and other inflammatory cytokines that cause systemic inflammation. Such release of pro-inflammatory cytokines including IL-1, IL-6, and tumor necrosis factor-α (TNF-α) may increase osteoclasts activation, thus disrupting the equilibrium between bone resorption and bone formation and ultimately leading to bone loss [16,17,18]. The inflammatory cytokines are also important stimulators of the receptor activator of nuclear factor-kB ligand (RANKL) synthesis, and its overwhelming production during the inflammatory process exceeds the production of its physiologic inhibitor and decoy receptor osteoprotegerin (OPG). The imbalance of RANKL/OPG ratio is directly responsible for bone loss in RA and other inflammatory diseases. Inflammatory cytokines can also influence osteoblastic function [19]. Therefore, chronic inflammatory diseases such as RA are considered to be a risk factor for osteoporosis and fractures [20]. However, unlike the well-known link between another inflammatory arthritis, rheumatoid arthritis (RA), and osteoporosis [20,21,22,23], conflicting data exist with regard to the association between gout and the risk of osteoporotic fractures. While some epidemiologic studies suggest that gout should be considered as a risk factor for fractures [24,25,26], others show no association between gout and osteoporotic fracture [27]. Furthermore, a recent meta-analysis presented that gout was not associated with an increased risk of fractures compared with a control group [28], while another meta-analysis revealed that gout was associated with an increased risk of any type of fracture as well as osteoporotic fractures [29]. Although it is possible that gout has a detrimental effect on osteoporosis through hyperuricemia or inflammation, other characteristics of gout patients, including middle-aged male predominance and obesity, could counter act.

There is a well-known link between RA and fracture risk, and some similarities exist between RA and gout such as inflammatory arthritis, bone erosion, and the use of oral steroids. Therefore, we aimed to evaluate the risk of non-vertebral or hip fractures in patients with gout compared to those with RA in a nationally representative cohort of older adults enrolled in Medicare. We hypothesized that older patients with gout would have a similar risk of osteoporotic fracture compared with older patients with RA.

## 2. Materials and Methods

### 2.1. Data Source

We conducted a cohort study using longitudinal claims data from Medicare (Parts A, B, and D) for the period between January 1, 2008 and September 30, 2015. Medicare is a US public health plan that primarily insures all Americans aged 65 years and older. Some younger individuals with certain disabilities are also included in Medicare. Medicare provides coverage for care received in hospitals (Part A), in physician’s offices or outpatient settings (Part B), and prescription medications (Part D). This study protocol was approved by the Institutional Review Board of the Brigham and Women’s Hospital (IRB Protocol No. 2014P001971) which waived the requirement for obtaining informed consent.

### 2.2. Study Cohort

**Figure 1:**
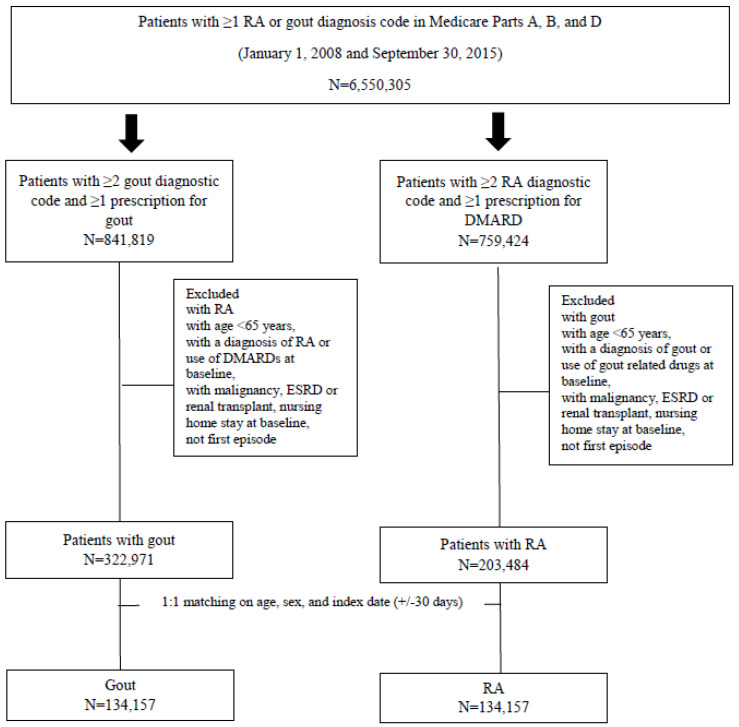
Flow diagram of cohort selection. RA: rheumatoid arthritis, DMARDs: disease modifying anti-rheumatic drugs, ESRD: end stage renal disease.

Among a total of over 5 million patients with a diagnosis code for gout or RA in Medicare, we selected the gout group based on ≥2 diagnosis codes separated by 7–365 days for gout and ≥1 dispensing for gout-related treatment (i.e., allopurinol, febuxostat, colchicine, probenecid, non-steroidal anti-inflammatory drugs (NSAIDS), and steroids). For the comparison, we identified the RA group with ≥2 diagnosis codes separated by 7–365 days and ≥1 dispensing for disease-modifying antirheumatic drugs (DMARDs) within a period of 1 year from the second RA diagnosis date. For both groups, the index date was the first dispensing date of gout or RA medication after the 2nd diagnosis date. All patients were required to be continuously enrolled in Medicare Parts A, B, and D for at least 1 year prior to the index date. All patients were required to be ≥65 years old at the index date. We excluded patients with malignancy, end-stage renal disease or renal transplantation in the baseline 365-day period. We also excluded patients who had a nursing home stay in the baseline 365-day period because of the lack of drug dispensing data during a nursing home stay. For gout patients, those with an RA diagnostic code or DMARDs, and for RA patients, those with a gout diagnostic code or gout medications in the baseline period were excluded. Gout patients were matched to RA patients on age, sex, and index date with a 1:1 ratio (Figure 1). Follow-up time started from the day after the index date to the occurrence of the outcomes, death, the disenrollment from insurance plan, or the end of the database, whichever came first.

### 2.3. Outcome Definition

The primary outcome of interest was non-vertebral fractures, a composite endpoint of humerus, wrist, pelvis, or hip fracture, based on previously validated claims-based algorithms that used ICD-9 diagnostic and procedure codes as well as current procedural terminology (CPT)-4 codes [30,31]. These algorithms were found to have a positive predictive value over 93%. The secondary outcome was hip fractures based on a combination of inpatients’ ICD-9 diagnostic codes for hip fracture and procedure or CPT-4 codes for hip fracture-related procedure or surgery [30,31]. We also assessed all-cause death.

### 2.4. Covariate Assessment

We assessed 45 baseline variables potentially related to presence of gout or RA and development of osteoporosis and/or fracture. These include age, sex, race, known risk factors for osteoporosis or fracture (i.e., prior BMD test, fall, history of fracture, obesity, and osteoporosis, use of osteoporosis-related medication, and prior steroids use) and other comorbidities. We estimated a comorbidity score that combined conditions in the Charlson and Elixhauser index [32] and included a claims-based frailty index [33] to account for baseline frailty in the study groups. In addition, we assessed other medications and markers of health care utilization intensity. All covariates were assessed during the baseline 365-day period, except the prior fracture and hip fracture variables, which were based on all available time before the index date in the database.

### 2.5. Statistical Analysis

The baseline characteristics of gout and RA patients were described with mean (standard deviation, SD) or number (percentage). We used the standardized difference to assess the balance of baseline covariates between the two groups; the standardized difference greater than 10% suggests a significant imbalance in the covariates between the two groups [34]. We estimated the incidence rate (IR) per 1000 person-year (PY) with 95% confidence interval (CI) and incidence rate ratio (IRR) of the primary and secondary outcomes in each group. We also performed stratified analysis by age, sex, prior fracture, cumulative dose of steroid, and no use of TNF inhibitors. Multivariable Cox proportional hazards models were used to estimate the hazard ratio (HR) with 95% CI for the primary and secondary outcomes in gout versus RA patients. Model 1 was adjusted for age and sex. Model 2 was further adjusted for calendar year, osteoporosis or fracture risk factors including prior BMD test, fall, prior fracture, obesity, osteoporosis medication use, and use and dose of steroids plus the Model 1 variables. Model 3 was further adjusted for race, frailty, comorbidity index, and the number of prescription drugs plus the variables included in the Model 2. Our final model included all the aforementioned 45 baseline covariates. We tested the interaction between gout and sex on the risk of any fracture and hip fracture and found no significant interaction by sex (*p* = 0.86 in any fracture and *p* = 0.61 in hip fracture). The proportional hazards assumption was assessed by testing the significance of the interaction term between exposure and time and was not violated in any of the Cox models. All analyses were performed using SAS 9.4 Statistical Software (SAS Institute Inc., Cary, NC, USA).

## 3. Results

### 3.1. Patient Characteristics

We included a total of 134,157 pairs of gout and RA patients matched on age, sex, and index date. The mean (SD) age was 73.7 (6.5) years and 70.4% were female in both groups (Table 1). Risk factors for osteoporotic fracture such as White race, prior BMD test, diagnosis of osteoporosis, use of osteoporosis medication, prior steroid use, and cumulative dose of steroid were more prevalent in RA than gout. However, other comorbidities including obesity, coronary heart disease, heart failure, hypertension, hyperlipidemia, diabetes, and chronic kidney disease were more frequently noted in patients with gout than RA. Gout patients had a higher comorbidity score and frailty index than RA patients.

### 3.2. Risk of Non-Vertebral Fracture, Hip Fracture and All-Cause Death

Over the mean 2.8 years of follow-up, non-vertebral fractures occurred in 3891 patients with gout and 5785 with RA with the corresponding IR per 1000 PY of 10.42 (95% CI 10.10–10.75) in gout and 15.01 (95% CI 14.63–15.40) in RA (Table 2). The IR per 1000 PY of hip fracture was 4.86 (95% CI 4.64–5.09) in gout and 7.73 (95% CI 7.46–8.01) in RA. The rate of all-cause mortality per 1000 PY was 19.96 in gout and 16.80 in RA.

Table 3 summarizes our results from multivariable Cox regression models. Age- and sex-adjusted HR (Model 1) associated with gout versus RA was 0.69 (95% CI 0.66–0.72) for non-vertebral fractures and 0.62 (95% CI 0.59–0.66) for hip fractures. The HR (Model 1) of all-cause death of gout versus RA was 1.18 (95% CI 1.14–1.22). The fully adjusted final model showed a decreased risk of non-vertebral fractures (HR 0.84, 95% CI 0.80–0.88) or hip fractures (HR 0.76, 95% CI 0.71–0.82) in gout compared with RA. In the final model, the risk of all-cause death was not different in gout compared with RA (HR 0.99, 95% CI 0.95–1.03).

Final model: All other comorbidities (smoking, alcoholism, dementia, Parkinson, coronary heart diseases, chronic obstructive pulmonary disease, diabetes, heart failure, hypertension, hyperlipidemia, chronic kidney diseases, stroke), medication use (TNF inhibitors, DMARDs, ACE inhibitors, anticonvulsants, antipsychotic agents, benzodiazepines, beta blocker, calcium channel blocker, diuretics, NSAIDs, opioids, proton pump inhibitor, selective serotonin reuptake inhibitor, statin, injection with steroid), and intensity of health care utilization (number of ER visits, OPD visits, and hospitalizations) in addition to model 3.

### 3.3. Stratified Analysis

Table 2 presents stratified IR and IRR by age, sex, prior fracture, 365-day cumulative dose of steroids, and absence of TNF inhibitor use. In both groups, the IRs of non-vertebral fractures and hip fractures were two to three times greater in patients aged ≥75 years compared with patients aged 65–74 years. As expected, the IR of non-vertebral fractures and hip fractures were more than two-fold higher in women than in men in both groups. The IRs of non-vertebral fractures and hip fracture were nearly three-fold higher in the patients with prior fracture than the patients without prior fracture in gout and RA. The IRs of non-vertebral fractures and hip fractures were slightly higher in the subgroups with a higher cumulative dose of steroids. Regardless of subgroups, IRRs for non-vertebral fractures and hip fractures were all lower for gout versus RA.

**Figure 2:**
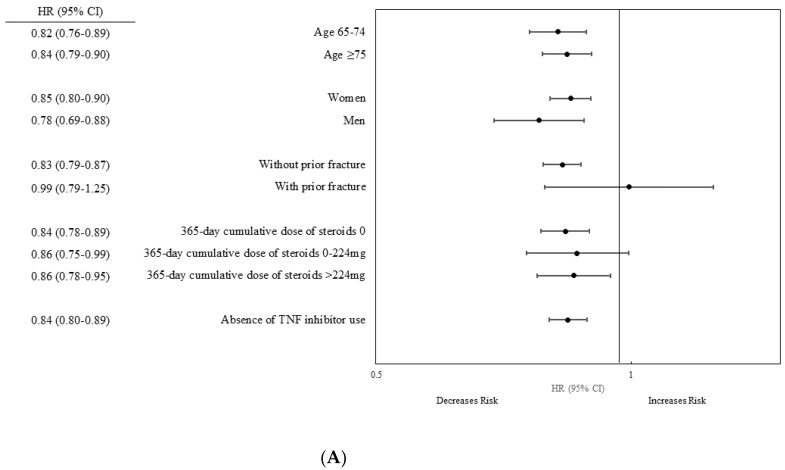

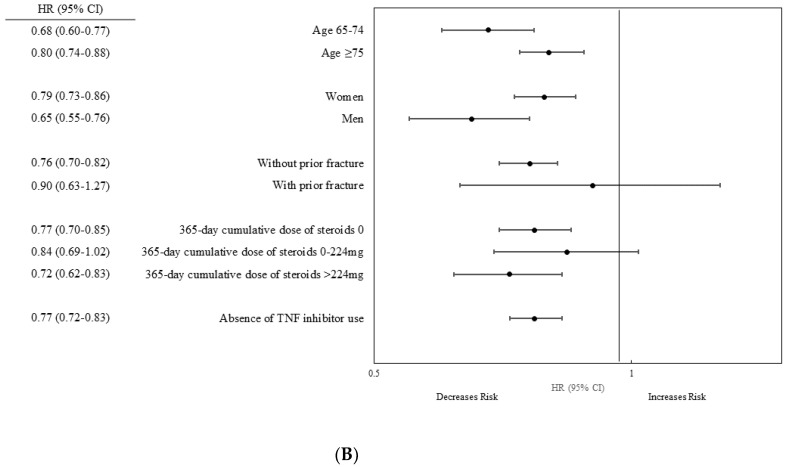
Stratified analysis: Multivariable hazard ratios of fracture in the gout group compared with the RA group (**A**). Non-vertebral fracture, (**B**). Hip fracture.

Figure 2 illustrates the results from the stratified multivariable Cox regression models (Final model). The HR for non-vertebral fractures and hip fractures in gout were consistently lower versus RA after stratifying by age, sex, 365-day cumulative dose of steroids, and absence of TNF inhibitor use. However, among patients with a prior fracture, the risk of non-vertebral fractures (HR 0.99, 95% CI 0.79–1.25) and hip fractures (HR 0.90, 95% CI 0.63–1.27) was similar between the two groups.

## 4. Discussion

Until now, the information on the risk of fracture associated with gout has been limited, although some studies have suggested a protective effect of uric acid on BMD or the risk of osteoporotic fracture [8,9,10]. Despite the well-known link between RA and fracture risk, and some similarities between RA and gout such as inflammatory arthritis, bone erosion, and the use of oral steroids, no studies have examined the risk of non-vertebral fractures or hip fractures in patients with gout compared to RA.

This large cohort study of older adults enrolled in Medicare found that nearly 2.9% of gout and 4.3% of RA patients developed non-vertebral fractures during the mean 2.8 years of follow-up. Both absolute and relative risk of non-vertebral and hip fractures was lower in patients with gout compared to RA, whereas all-cause mortality was similar between the two groups. It is interesting to note that both groups had many comorbidities, but they exhibited different patterns of comorbidities associated with fractures; more RA patients had osteoporosis and used osteoporosis medications and steroids, while more gout patients were obese, frail, and had greater comorbidities. Such variation in the patterns of comorbidities between gout and RA could, in part, explain the observed lower risk of non-vertebral fractures and hip fractures in gout compared with RA patients in our study. Another important distinction between the two diseases is the sex difference. Unlike gout affecting more men than women, RA is more common in women; this difference could potentially explain a lower rate of fractures in gout versus RA as female sex is a risk factor for osteoporosis and fracture [35]. However, our patients were matched on sex. Furthermore, we found a lower risk of fracture in gout compared to RA even after stratifying by sex.

Gout is an inflammatory disease leading to bone erosion; however, unlike RA, gout is an arthritis with episodic flares causing intense local inflammation. Although local inflammation that needs an adequate supply of calcium and phosphorus can increase the local bone turnover [36], the association between local inflammation and systemic bone turnover has not been well-studied. It is possible that persistent systemic and/or local inflammation seen in RA contributes to osteoporosis or fracture much more than episodic, albeit intense, inflammation caused by gout.

Our study has several strengths. First, we included a nationally representative large cohort of older adults at high risk for fracture. Therefore, compared to previous studies, our result showed a higher IR of non-vertebral fractures and hip fractures in patients with gout—10.42/1000 PY and 4.86/1000 PY, respectively. One observational study using claims data including a population with a mean age of 60 years and 18% women presented that the IR of non-vertebral fractures and hip fractures in gout was 2.92/1000 PY and 0.86/1000 PY, respectively [27]. Similarly, in a prospective cohort study using the Nurses’ Health Study, the IR of hip fractures was 2.34/1000 PY in women with gout and the mean age of their female population was 59.6 [25]. Another claims data-based cohort study of middle-aged patients (mean age 50.9 years, 30.1% women) also showed the IR of hip fractures in gout as 1.54/1000 PY [24]. After stratifying by age and sex, regardless of having gout or RA, we noted that the IR of non-vertebral fractures and hip fractures were more than two-fold higher in patients aged ≥75 years than patients aged 65–74 years, and those were more than two-fold higher in women than in men. Third, we used the validated claims-based algorithms to define non-vertebral fractures to minimize outcome misclassification [30,31]. Fourth, even though the fracture risk in patients with gout was lower relative to that of RA patients, we showed a high absolute rate of non-vertebral fractures in patients with gout and a low proportion of gout patients being managed for osteoporosis in a real-world setting. With a significant increase in the life expectancy of the overall population, this present study underscores the importance of osteoporosis or fracture prevention/management in older patients with gout as well as RA. Furthermore, among patients with a prior fracture, gout and RA patients had a similar risk of non-vertebral fractures. This finding emphasizes the importance of secondary prevention of osteoporotic fractures in older patients regardless of their underlying type of arthritis.

There are limitations in our study. First, we were unable to account for the disease (i.e., gout or RA) severity and duration as such information was not available in our database. It is possible that gout flare has a different impact on the risk of fall or fracture compared with patients with chronic gouty arthritis. Second, we could not assess the impact of serum urate levels on osteoporotic fractures because of the lack of laboratory results in our database. Third, we did not assess vertebral fractures as it is difficult to accurately capture the onset of vertebral fractures in claims data. Fourth, while our Final models were adjusted for over 45 variables, there may be residual confounding by unmeasured factors including body mass index, BMD, diet pattern, nutritional status, and physical activity. Fifth, patients with gout in our study were older and more women than those with general gout. We examined the risk of fracture associated with gout in older patients compared to age- and sex- matched patients with RA. Therefore, careful interpretation of the fracture risk of gout is required. Lastly, our baseline period of 365 days prior to the index date may not be sufficiently long enough to capture patients’ baseline characteristics.

## 5. Conclusions

In this large cohort of older patients with the mean age over 73 enrolled in Medicare, gout was associated with a decreased risk of non-vertebral and hip fractures regardless of age, sex, steroid use, and prior fracture, compared to RA. However, the absolute rate of non-vertebral fractures or hip fractures was high in both gout and RA patients and all-cause mortality was not different between the two groups. Our results highlight the importance of patient and physician education with regard to preventing and managing osteoporosis and fractures in older patients with not only RA but also gout.

## Figures and Tables

**Table 1:** Baseline characteristics of study populations in the 365 days before study entry.

Variable	Gout (*n* = 134,157)	RA (*n* = 134,157)	Standardized Difference * (%)
Demographics ^†^			
Age, year, mean ± SD	73.69 ± 6.52	73.69 ± 6.52	0.0
Female	94,456 (70.41)	94,456 (70.41)	0.0
White race	99,924 (74.48)	115,625 (86.19)	29.8
Osteoporotic fracture related comorbidities			
BMD test	13,922 (10.38)	33,274 (24.80)	38.6
Prior fall	5953 (4.44)	5810 (4.33)	−0.5
Osteoporosis	16,654 (12.41)	44,135(32.90)	50.5
Obesity	23,598 (17.59)	9802 (7.31)	−31.5
Prior fracture ^‡^	2649 (1.97)	3426 (2.55)	3.9
Prior hip fracture ^‡^	753 (0.56)	1246 (0.93)	4.3
Other comorbidities			
Comorbidity score, mean ± SD	2.03 ± 2.66	1.03 ± 1.93	−0.4
Smoking	13,539 (10.09)	13,277 (9.90)	−0.6
Alcoholism	581 (0.43)	278 (0.21)	−3.9
Dementia	8879 (6.62)	8172 (6.09)	−2.2
Parkinson	1602 (1.19)	1772 (1.32)	1.2
Coronary heart diseases	55,635 (41.47)	39,839 (29.70)	−24.8
COPD	38,012 (28.33)	35,979 (26.82)	−3.4
Diabetes mellitus	67,909 (50.62)	37,643 (28.06)	−47.5
Heart failure	33,290 (24.81)	14,758 (11.00)	−36.6
Hypertension	126,632 (94.39)	101,379 (75.57)	−54.6
Hyperlipidemia	115,472 (86.07)	95,978 (71.54)	−36.1
Chronic kidney diseases	46,420 (34.60)	13,829 (10.31)	−60.8
Stroke	24,349 (18.15)	18,855 (14.05)	−11.2
Frailty index			
Robust	35,902 (26.76)	41,942 (31.26)	9.9
Prefrail	81,216 (60.54)	81,327 (60.62)	0.2
Mildly frail	15,975 (11.91)	10,389 (7.74)	−14.0
Moderately to severely frail	1064 (0.79)	499 (0.37)	−5.5
Osteoporosis medication			
Bisphosphonate	10,061 (7.50)	29,866 (22.26)	42.4
PTH	191 (0.14)	941 (0.70)	8.7
Calcitonin	573 (0.43)	1183 (0.88)	5.6
Denosumab	411 (0.31)	1401 (1.04)	8.9
Raloxifene	1640 (1.22)	2294 (1.71)	4.1
Teriparatide	191 (0.14)	941 (0.70)	8.7
Other medications			
Prior use of steroids	41,047 (30.60)	74,294 (55.38)	−51.7
Prior cumulative dose of prednisolone equivalent, mg, mean ± SD	160.57 ± 540.52	673.19 ± 1096.69	0.59
0	93,110 (69.40)	59,863 (44.62)	−51.7
>0 and ≤224	20,503 (15.28)	12,786 (9.53)	−17.5
>224	20,544 (15.31)	61,508 (45.85)	70.3
Steroid injection	10,357 (7.72)	18,893 (14.08)	20.5
TNF inhibitor	0(0)	13,061 (9.74)	46.5
Methotrexate	0(0)	83,428 (62.19)	181.4
Anticonvulsants	21,499 (16.03)	22,030 (16.42)	1.1
Antipsychotic	32,739 (24.40)	32,007 (23.86)	−1.3
Benzodiazepines	15,823 (11.79)	17,571 (13.10)	4.0
Beta blocker	62,397 (46.51)	44,015 (32.81)	−28.3
ACE inhibitor	91,489 (68.20)	63,783 (47.54)	−42.8
Calcium channel blocker	54,211 (40.41)	37,065 (27.63)	−27.2
Diuretics	97,994 (73.04)	58,219 (43.40)	−63.0
Statin	80,794 (60.22)	61,059 (45.51)	−29.8
NSAIDs	64,465 (48.05)	58,786 (43.82)	−8.5
Opioids	58,529 (43.63)	56,567 (42.16)	−3.0
Proton pump inhibitor	44,834 (33.42)	48,896 (36.45)	6.4
SSRI	21,975 (16.38)	26,365 (19.65)	8.5
Health care utilization, mean ± SD			
No. of all prescription drugs	13.60 ± 6.49	12.08 ± 6.11	−0.2
No. of ER visit	0.86 ± 1.62	0.52 ± 1.12	−24.1
No. of visits	12.95 ± 9.16	14.21 ± 9.17	0.1
No. of hospitalization	0.41 ± 0.87	0.24 ± 0.61	−20.4

Data are presented as number (%). * A standardized difference of 10% denotes a meaningful imbalance in the covariates between gout and RA (34). ^†^ Gout and RA groups are age, sex, and index date matched. **^‡^** Prior fracture means fracture during any time periods before the start of follow-up. RA: rheumatoid arthritis, SD: standardized deviation, BMD: bone mineral density, COPD: chronic obstructive pulmonary disease, PTH: parathyroid hormone, TNF: tumor necrosis factor, ACE: angiotensin converting enzyme, NSAIDs: non-steroidal anti-inflammatory drugs, SSRI: selective serotonin reuptake inhibitors, ER: emergency room.

**Table 2:** Incidence rates and incidence rate ratio of fracture per 1000 person-year in the gout group versus RA group: age, sex, and index date matched.

		Gout Group				RA Group				IRR (95% CI)
	Subgroup	No. of Patients	Cases	Person-Years	IR (95% CI)	No. of Patients	Cases	Person-Years	IR (95% CI)	
Non-vertebral fracture										
All patients		134,157	3891	373,336	10.42 (10.10–10.75)	134,157	5785	385,306	15.01 (14.63–15.40)	0.69 (0.66–0.72)
Age, years	65–74	81,439	1642	235,612	6.97 (6.64–7.32)	81,439	2570	246,103	10.44 (10.04–10.85)	0.67 (0.63–0.71)
	≥75	52,718	2249	137,723	16.33 (15.67–17.02)	52,718	3215	139,202	23.10 (22.32–23.91)	0.71 (0.67–0.75)
Sex	Women	94,456	3258	254,914	12.78 (12.35–13.23)	94,456	4854	263,895	18.39 (17.88–18.91)	0.69 (0.66–0.72)
	Men	39,701	633	118,421	5.35 (4.95–5.78)	39,701	931	121,410	7.67 (7.19–8.18)	0.70 (0.63–0.77)
Prior fracture	No	131,508	3701	368,057	10.06 (9.74–10.39)	130,731	5501	378,326	14.54 (14.16–14.93)	0.69 (0.66–0.72)
	Yes	2649	190	5278	35.99 (31.22–41.49)	3426	284	6979	40.69 (36.22–45.71)	0.88 (0.73–1.06)
365-d cumulative dose of steroids	0	93,110	2561	264,162	9.69 (9.32–10.07)	59,863	2473	177,812	13.91 (13.37–14.47)	0.70 (0.66–0.74)
	0–224 mg	20,503	634	56,134	11.29 (10.44–12.20)	12,786	527	36,930	14.27 (13.10–15.54)	0.79 (0.70–0.89)
	>224 mg	20,544	696	53,039	13.12 (12.18–14.13)	61,508	2785	170,563	16.33 (15.73–16.95)	0.80 (0.74–0.87)
TNF inhibitor	No	134,157	3891	373,336	10.42 (10.10–10.75)	121,096	5270	351,257	15.00 (14.60–15.41)	0.69 (0.66–0.72)
	Yes					13,061	515	34,048	15.13 (13.88–16.49)	NA
Hip fracture										
All patients		134,157	1831	376,535	4.86 (4.64–5.09)	134,157	3013	389,675	7.73 (7.46–8.01)	0.63 (0.59–0.67)
Age, years	65–74	81,439	588	237,454	2.48 (2.29–2.69)	81,439	1142	248,645	4.59 (4.33–4.86)	0.54 (0.49–0.60)
	≥75	52,718	1243	139,081	8.94 (8.46–9.45)	52,718	1871	141,029	13.27 (12.68–13.89)	0.67 (0.62–0.72)
Sex	Women	94,456	1492	257,622	5.79 (5.50–6.09)	94,456	2439	267,678	9.11 (8.76–9.48)	0.64 (0.60–0.68)
	Men	39,701	339	118,913	2.85 (2.56–3.17)	39,701	574	121,996	4.71 (4.34–5.11)	0.61 (0.53–0.70)
Prior fracture	No	131,508	1755	371,107	4.73 (4.51–4.96)	130,731	2880	382,493	7.53 (7.26–7.81)	0.63 (0.59–0.67)
	Yes	2649	76	5428	14.00 (11.18–17.53)	3426	133	7181	18.52 (15.63–21.95)	0.76 (0.57–1.01)
365-d cumulative dose of steroids *	0	93,110	1218	266,342	4.57 (4.32–4.83)	59,863	1257	179,791	6.99 (6.61–7.39)	0.65 (0.60–0.70)
	0–224 mg	20,503	304	56,640	5.37 (4.80–6.01)	12,786	263	37,300	7.05 (6.25–7.96)	0.76 (0.64–0.90)
	>224 mg	20,544	309	53,552	5.77 (5.16–6.45)	61,508	1493	172,583	8.65 (8.22–9.10)	0.67 (0.59–0.76)
TNF inhibitor	No	134,157	1831	376,535	4.86 (4.64–5.09)	121,096	2750	355,227	7.74 (7.46–8.03)	0.63 (0.59–0.67)
	Yes					13,061	263	34,447	7.63 (6.76–8.61)	NA

RA: rheumatoid arthritis, IRR: incidence rate ratio, CI: confidence interval, * prednisolone equivalent dose.

**Table 3:** Risk of non-vertebral or hip fracture in the gout group compared with the RA group.

Adjustment	Hazard Ratio (95% CI)
Non-vertebral fracture	
Model 1	0.69 (0.66–0.72)
Model 2	0.78 (0.75–0.82)
Model 3	0.75 (0.72–0.79)
Final model	0.84 (0.80–0.88)
Hip fracture	
Model 1	0.62 (0.59–0.66)
Model 2	0.72 (0.67–0.76)
Model 3	0.68 (0.64–0.73)
Final model	0.76 (0.71–0.82)

Model 1: Age, sex-adjusted. Model 2: Calendar year, bone mineral density test, Fall, obesity, prior fracture, bisphosphonates, use of non-bisphosphonate osteoporosis medications, recent use of steroids, and 365-day cumulative dosage of steroids in addition to model 1. Model 3: Race, frailty index, comorbidity index, and number of different prescriptions in addition to model 2.

## Data Availability

All relevant data are within the paper.
